# Effects of Shaoyao-Gancao Decoction on Infarcted Cerebral Cortical Neurons: Suppression of the Inflammatory Response following Cerebral Ischemia-Reperfusion in a Rat Model

**DOI:** 10.1155/2016/1859254

**Published:** 2016-06-20

**Authors:** Ying Zhang, Xinling Jia, Jian Yang, Qing Li, Guofeng Yan, Zhongju Xu, Jingye Wang

**Affiliations:** ^1^Department of Rehabilitation, Shanghai Xuhui Central Hospital, Shanghai Clinical Center, Chinese Academy of Sciences, No. 966, Huaihai Road, Shanghai 200031, China; ^2^School of Life Sciences, Shanghai University, No. 99 Shangda Road, Shanghai 200444, China; ^3^School of Medicine, Jiao Tong University, No. 280, Chongqing South Road, Shanghai 200025, China; ^4^Department of Chinese Medicine, Shanghai Punan Hospital, No. 519, Pudong New Area South Wharf Road, Shanghai 200125, China; ^5^Department of Rehabilitation, Shanghai Dahua Hospital, No. 903, Old Humin Road, Xuhui District, Shanghai 200237, China

## Abstract

The mechanisms by which Shaoyao-Gancao decoction (SGD) inhibits the production of inflammatory cytokines in serum and brain tissue after cerebral ischemia-reperfusion (CI-RP) in rats were investigated. A right middle cerebral artery occlusion was used to induce CI-RP after which the rats were divided into model (*n* = 39), SGD (*n* = 28), clopidogrel (*n* = 25) and sham operated (*n* = 34) groups. The Bederson scale was used to evaluate changes in behavioral indices. The levels of IL-1*β*, TNF-*α*, MCP-1, IL-10, RANTES, VEGF, and TGF-*β*1 in the serum and infarcted brain tissues were measured. Nissl body and immunohistochemical staining methods were used to detect biochemical changes in neurons, microglial cells, and astrocytes. Serum levels of VEGF, TNF-*α*, MCP-1, IL-1*β*, and IL-10 increased significantly 24 h after CI-RP. In brain tissue, levels of TNF-*α* and IL-1*β* significantly increased 24 h after CI-RP, whereas levels of TGF-*β*1 and MCP-1 were significantly higher 96 h after CI-RP (*P* < 0.05). SGD or clopidogrel after CI-RP reduced TNF-*α* and IL-1*β* levels in brain tissue and serum levels of MCP-1, IL-1*β*, and IL-10. SGD increased the number of NeuN-positive cells in infarcted brain tissue and reduced the number of IBA1-positive and GFAP-positive cells. The efficacy of SGD was significantly higher than that of clopidogrel.

## 1. Introduction

The severity of brain injury caused by cerebral ischemia increases as the duration of the ischemic period increases prior to reperfusion. Many researchers believe that the early immunoinflammatory response following cerebral ischemia-reperfusion (CI-RP) is an important factor in ischemia-induced neuronal apoptosis [1] and that circulating regulatory T cells can reduce long-term lymphopenia and improve postischemia cellular immunity in the brain. Previous studies have shown that animals treated with regulatory T cells exhibit reduced bacterial loads and inflammatory response during recovery from a cerebral ischemic event [2].

The inflammatory response after CI-RP may not be limited to brain tissues because the level of inflammatory factors in serum dramatically increases as a result of damage to the blood brain barrier [3]. Several hours after CI-RP, microglial cells, astrocytes, and intrinsic cells can be activated to produce proinflammatory factors such as tumor necrosis factor- (TNF-) *α* [4] and interleukin- (IL-) 1*β* [5] and chemotactic factors such as monocyte chemoattractant protein-1 (MCP-1) [6]. Therefore, reducing the post-CI-RP inflammatory response can reduce the severity of cerebral injury [7]. In China, antiplatelet drugs are routinely administered following acute cerebral infarction. Clopidogrel inhibits platelet aggregation by binding to the adenosine diphosphate (ADP) receptor on platelet membranes [8]. Clopidogrel also inhibits platelet activation induced by other aggregation agonists by blocking ADP binding [9], and treatment with clopidogrel and aspirin is more effective for inhibiting platelet activation than aspirin alone [9].

The Shaoyao-Gancao decoction (SGD) is an extract of the plants,* Paeonia lactiflora* and* Glycyrrhiza uralensis*, which is used in traditional Chinese medicine. Paeoniflorin is the main active ingredient of SGD. The cerebral protective effects of paeoniflorin are mediated by improvement in mitochondrial dysfunction [10].* In vitro* experiments have shown that paeoniflorin protects against neuronal loss by inhibiting TNF-*α*-induced apoptosis, and paeoniflorin has also been shown to inhibit MAPK/NF*κ*B-mediated cerebral and peripheral inflammatory responses in a rat model of CI-RP [11]. Nam and colleagues showed that paeoniflorin inhibits lipopolysaccharide-induced apoptosis of hippocampal neurons by reducing the expression of nitric oxide and IL-1*β* in the hippocampus [12]. Paeoniflorin also reduces the secretion of proinflammatory factors from microglial cells, such as nitric oxide, IL-1*β*, and TNF-*α*, the inhibition of which is thought to mediate the major neuroprotective effects of paeoniflorin.

Although previous studies have investigated the effects of paeoniflorin on the immune-inflammatory response after cerebral injury, a comprehensive analysis of the effects of SGD on both the cerebral and systemic immune-inflammatory responses following cerebral injury has not been performed. Furthermore, the effects of SGD on infarcted cerebral cortical neurons and neurobehavioral indices following CI-RP have not been reported. In the present study, we examined the effects of SGD on neurobehavioral indices and the cerebral and systemic proinflammatory cytokine profiles of rats following CI-RP and compared them in rats treated with clopidogrel to characterize the anti-inflammatory and neuroprotective effects of SGD.

## 2. Material and Methods

### 2.1. Animals

Specific-pathogen-free 8-week-old male Sprague-Dawley rats weighing 250 to 300 g (Slack Experimental Animal Company, Shanghai, China) were housed in separate cages in the Animal Facility of the Animal Science Department of the Medical College of Shanghai, Jiao Tong University. The rats were acclimatized for 1 week at 23°C with a 12:12 h dark/light cycle, and food and water were provided* ad libitum*. Our study protocol was approved by the Animal Ethics Committee of the Medical College of Shanghai, Jiao Tong University (approval number 2013102).

### 2.2. SGD Preparation

SGD containing 1 g of Radix Paeoniae Alba (Cambridge Chinese Herbal Medicine, Shanghai, China) and 1 g of Radix Glycyrrhizae Preparata (Tongjitang Chinese Herbal Medicine, Shanghai, China) was prepared according to a previous study [13] and provided by the TCM Preparation Room of the Longhua Hospital of Shanghai.

### 2.3. Model of CI-RP-Induced Brain Injury

The tip of a 2.2 cm 4-0 nylon thread was heated and stretched to a smooth spherical diameter of 2.2 to 2.4 mm. The thread was soaked in 75% alcohol before being immersed in a 1% heparin solution prior to embolism induction. Right middle cerebral artery occlusion (MCAO) was induced in 106 rats using the thread embolism method, as previously described by Kang et al. [14]. An additional 34 rats were assigned to the sham-surgery group but did not undergo the occlusion procedure. Each rat was fasted for the 12 h period preceding the experiment. An intraperitoneal injection of 350 mg·kg^−1^ chloral hydrate was administered to each rat to induce deep general anesthesia prior to surgery. After anesthesia was induced, the cervical region of each rat was shaved and the animal placed in the supine position. A 2 cm incision was made to expose the bilateral submandibular glands. The sternohyoid and bilateral sternocleidomastoid muscles were exposed by blunt dissection. The right mastoid muscles were separated by using microscopic hemostatic forceps to separate the sternohyoid muscle from the mucosa, and the right common carotid artery (CCA) was isolated using microscopic hemostatic forceps. A thread was used to position the vagus nerve along the course of the CCA and external carotid artery (ECA) to reduce respiratory secretions and to prevent asphyxia by stimulation of the trachea. A careful separation of the bifurcation of the distal CCA exposed the bifurcations of the ECA and internal carotid artery (ICA). The pterygopalatine artery was ligated and the ECA ligated near the bifurcation of the ECA and ICA, while the CCA was ligated close to its proximal end. A noninvasive, microscopic clip was used to occlude the ICA. An incision was made in the CCA 3 to 5 mm from the bifurcation of the ECA and ICA. A 1.6–1.8 cm thread embolism was inserted into the CCA and fixed in place to occlude the vessel, causing ischemia to the region of the brain supplied by the right middle cerebral artery. Two hours after ischemia was induced, reperfusion was initiated by removing the thread embolism and venous blood was collected for baseline serum cytokine profile assessments.

### 2.4. Evaluation of the MCAO Model

**Figure 1:**
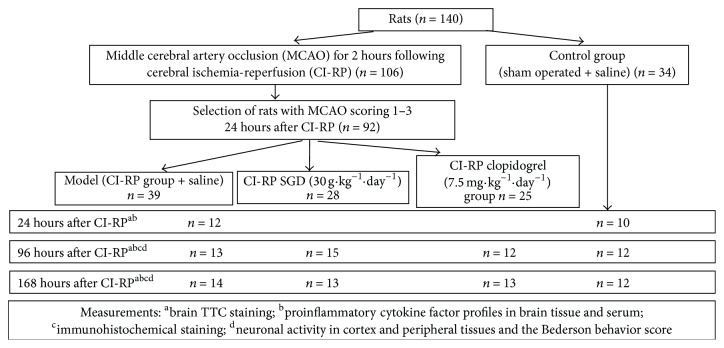
Flow chart of the present study.

The severity of CI-RP-induced brain injury was assessed at 24 h after surgery to characterize the resulting neurological deficits. All of the rats in the sham-surgery group remained living at 24 h after surgery. Each rat was scored on a scale from 0 to 4, based on the severity of the neurological deficits observed, as described previously [15]. A score of 0 was given if no manifestations of cerebral injury, such as hemiplegia or circling, were observed. A score of 1 was given to rats unable to extend their contralateral front and rear paws fully during locomotion. Rats that could walk but displayed involuntary circling to the left received a score of 2. Rats that could walk but displayed uncontrollable toppling to the left received a score of 3. Rats that were unable to walk or appeared unconscious were given a score of 4. Only rats that received a score of 1 to 3 were included in the SGD, clopidogrel, and model groups. Rats in the model (negative control) group (*n* = 39) and sham-surgery (positive control) group (*n* = 34) were given a gavage administration of 4 mL of normal saline once daily. The SGD group (*n* = 28) received a gavage dose of 30 g·kg^−1^·day^−1^ SGD and the clopidogrel group (*n* = 25) a gavage dose of 7.5 mg·kg^−1^·day^−1^ clopidogrel (Shenzhen Tailixin Pharmaceutical, Shenzhen, China). At each time point, 5 rats in each group underwent cerebral paraformaldehyde perfusion. The remaining rats were subsequently evaluated for neurological deficits at 96 h and 168 h after surgery, following the initiation of the experimental treatments (Figure 1). Venous blood was collected from each rat at 24, 96, and 168 h after CI-RP to analyze their serum cytokine profiles.

### 2.5. Evaluation of Neurological Behavior

Animal behavior was evaluated 24 h, 96 h, and 168 h after CI-RP based on a modified Bederson scoring method [16]. When a rat's tail was lifted, if the bilateral forelimbs were symmetrically stretched, the rat was given a score of 0. If wrist bending, elbow bending, shoulder internal rotation, or both wrist/elbow bending and shoulder internal rotation of the left forelimb were observed, the rat was given a score of 1, 2, 3, or 4, respectively. With rats supine, the shoulder was pulled to the left side to assess resistance. If the bilateral resistance was equivalent, the rat was given a score of 0. If progressively less resistance was observed when pulling to the left, the rat received a score of 1, 2, or 3 respectively, based on the level of resistance, with 1 indicating a slight difference and 3 indicating severe impairment in the left forelimb.

The forelimbs of rats were placed in a metal net to assess their grip strength. If the bilateral grip strengths were equivalent, the rat received a score of 0. Differences in bilateral grip strength were scored as 1, 2, or 3, with 1 indicating a slight difference and 3 indicating severe impairment in the left forelimb. Rats that circled continuously to the left during locomotion were given a score of 1. According to standard scoring, the highest possible score was 11, with higher scores indicating more serious behavioral dysfunction.

### 2.6. Brain Tissue Collection

Rats underwent cerebral paraformaldehyde perfusion before their brains were harvested, described in a previous study [17]. After general anesthesia was induced intraperitoneally with 10% chloral hydrate, the abdomen was incised. Surgical scissors were used to open the diaphragm and break the bilateral ribs up to the infraclavicular plexus. The diaphragm was lifted and fixed in place. The ascending aorta was separated and a 16-gauge needle was inserted into the ascending aorta, extending through the left ventricle to the apex of the heart, and then hemostatic forceps were attached to the cardiac muscle. Surgical scissors were used to open the atrial appendage and the infusion was initiated using a controlled pressure range between 160 and 200 mmHg. Approximately 100 mL of precooled normal saline was rapidly infused, during which the atrial appendage was flushed, followed by the rapid infusion of 100 mL of precooled 4% paraformaldehyde. The flow rate was reduced to a slow drip and an additional 100 mL of precooled 4% paraformaldehyde was slowly infused over a 30 min period. The brain was removed and immediately submerged in 4% paraformaldehyde for 24 h, after which it was submerged in a 30% sucrose solution. Brains were wrapped in silver paper and placed in liquid nitrogen before storage at −80°C. Pale tissues surrounding the infarct area were used for the analyses.

### 2.7. Assessment of the Infarction by 2,3,5-Triphenyltetrazolium Chloride (TTC) Staining of Rat Brains

**Figure 2:**
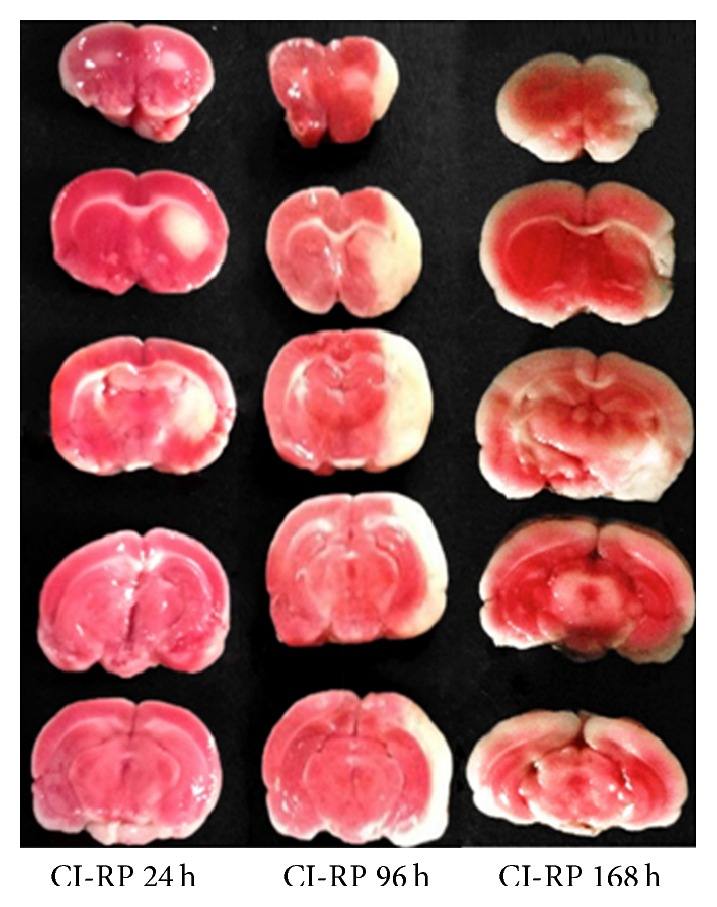
Assessment of successful infarction in rats of the model group at 24 (*n* = 1), 96 (*n* = 1), and 168 h (*n* = 1) after CI-RP, based on TTC staining.

One rat in the model group was humanely killed at 24, 96, and 168 h after CI-RP. The brain was removed and frozen immediately in liquid nitrogen before storage at −80°C. The cerebellum was removed from the brain 15 min after it was frozen. The whole brain was evenly sliced into 5 sections and soaked in 0.05% TTC staining at 37°C for 30 min in the absence of light, with turning every 5 min [18]. The size of the CI-RP-induced infarct and stability of the model were determined.  Figure 2 shows that the infarcted area was located in the corpus striatum and cerebral cortex at each time point, indicating excellent model stability.

### 2.8. Determination of Brain and Serum Cytokine Factor Profiles

After general anesthesia, venous blood was collected from the right atrial appendage of each rat. Samples of the cerebral cortex and corpus striatum tissues, surrounding the damaged areas, were also collected and placed in lysis buffer. The tissues were homogenized and the total protein concentration of the homogenate was determined using a BCA kit. The levels of IL-1*β*, IL-10, TNF-*α*, and TGF-*β*1 [19] as well as MCP-1 [20], vascular endothelial growth factor (VEGF) [21], and the regulated on activation normal T cell expressed and secreted (RANTES) [22] in the brain tissue homogenate and serum of rats were measured using the Bio-Plex Magpix System (Bio-Rad Laboratories, Hercules, CA, USA) according to the manufacturer's instructions.

### 2.9. Immunohistochemistry

Immunohistochemistry was performed to detect neuronal nuclear antigen (NeuN) as neuron marker [23], ionized calcium-binding adapter molecule 1 (IBA1) as microglia marker [24], and glial fibrillary acidic protein (GFAP) as astrocyte marker [25] in the brain tissues of rats. Frozen 25 *μ*m sections of fixed brain tissue were prepared. The murine anti-NeuN monoclonal antibody, goat anti-IBA1 polyclonal antibody, and murine anti-GFAP monoclonal antibody were each diluted 1 : 1000. The sections were incubated in these primary antibody solutions at room temperature, followed by incubation with biotin-labeled anti-goat Ig and anti-mouse Ig polyclonal secondary antibodies in 1 : 1000 dilution of horseradish peroxidase-labeled streptavidin. Staining was performed by incubation in a 0.03% solution of 3,3′-diaminobenzidine in phosphate-buffered saline, followed by incubation in 0.01% hydrogen peroxide for colour development. Cresyl violet staining of Nissl bodies was used for neuron-specific labeling. The stained sections were examined using light microscopy at ×400 magnification and the number of NeuN-positive, IBA1-positive, and GFAP-positive cells were quantified with IMAGE-PRO PLUS 6.0 software (Media Cybernetics Inc. Co., Shanghai, China).

### 2.10. Statistical Analysis

SPSS version 19.0 software (IBM, Armonk, NY, USA) was used for the statistical analysis. A sample size of 12 in each group was considered to provide 95% power for detecting MCP-1 expression level reductions (two-sided, *α* = 5%). To account for a 5% dropout, 140 rats in total (12 in each group) have been included. The distribution of each dataset was tested, and parametric (unpaired Student's* t*-test) or nonparametric (Mann-Whitney) tests were employed accordingly, while using ANOVA for sequential treatments, based on a Gaussian distribution. Continuous data are expressed as the mean ± standard deviation and categorical data (neurological behaviors) as the median and range. Generalized linear model (GLM) tests were used to compare continuous data for multiple groups and ANOVA or a rank-sum test to compare continuous data between two groups. The Wilcoxon rank-sum test was used to compare categorical data for multiple groups and the Student-Newman-Keuls (SNK) or ranked SNK test used to compare categorical data between two groups.

## 3. Results

### 3.1. Proinflammatory Cytokine Factor Profiles in Brain Tissue and Serum following CI-RP

The levels of proinflammatory cytokines in the brain tissue and serum of rats differed at the different time points following MCAO. The serum level of RANTES at 168 h after CI-RP was significantly reduced, compared to 24 h after CI-RP (*P* < 0.05) and the sham group (*P* < 0.01), whereas no change in the level of RANTES in brain tissue was detected after CI-RP. The level of VEGF in the serum increased significantly at 24 h after CI-RP (*P* < 0.05) and progressively decreased thereafter (*P* < 0.001). By contrast, the level of VEGF in brain tissue was equivalent to that of the sham-surgery group at all 3 time points. The levels of TNF-*α* in serum and brain tissue were similar, reaching a plateau at 24 h after CI-RP that was maintained at the 96 h and 168 h time points (*P* < 0.05) (*P* < 0.01). The level of TGF-*β*1 in brain tissue progressively increased until 168 h following CI-RP (*P* < 0.001), but the serum level of TGF-*β*1 was not significantly different than in the control group.

**Figure 3:**
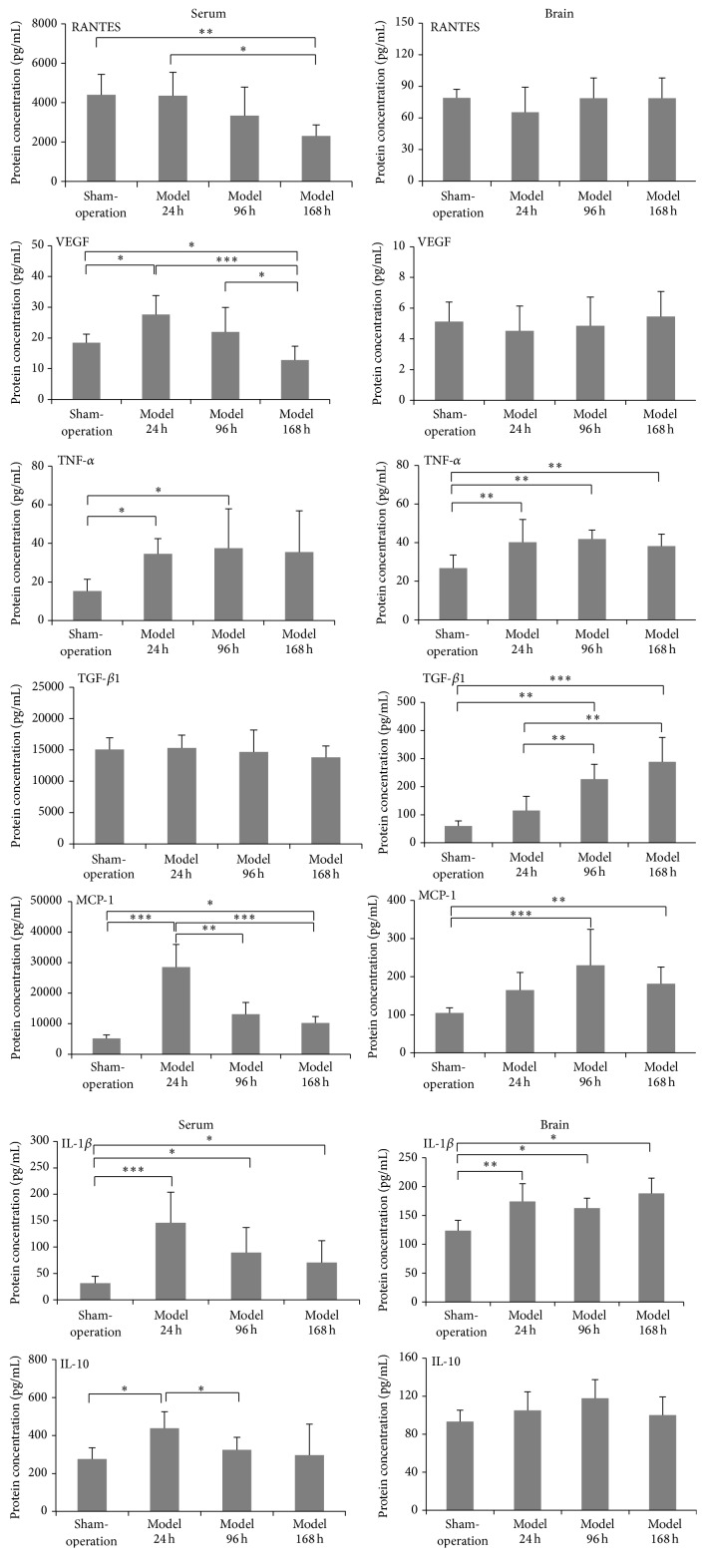
Changes in the levels of proinflammatory cytokines and angiogenic factors in the brain tissue and serum of rats at different time points following CI-RP. ^*∗*^ *P* < 0.05; ^*∗∗*^ *P* < 0.01; ^*∗∗∗*^ *P* < 0.001.

The serum level of MCP-1 at 24 h after CI-RP was significantly higher than that in the control group (*P* < 0.001) and was reduced to the control levels at 96 and 168 h after CI-RP (*P* < 0.01) (*P* < 0.001). The level of MCP-1 expression in brain tissue at 96 h after CI-RP was the highest (*P* < 0.01), thereafter decreasing. The expression of IL-1*β* in both serum and brain tissue rapidly increased to a maximum level at 24 h after CI-RP (*P* < 0.001) (*P* < 0.01) and declined thereafter in the serum at 96 h, whereas the concentration remained steady until 168 h after CI-RP in the brain tissue. The serum level of IL-10 rapidly increased at 24 h after CI-RP (*P* < 0.05) but returned to normal levels at 96 h (*P* < 0.05) and 168 h after CI-RP. No significant change in the level of IL-10 in brain tissue was detected following CI-RP. Therefore, with the exception of RANTES and TGF-*β*1, the highest levels of the proinflammatory cytokines, including VEGF, TNF-*α*, MCP-1, IL-1*β*, and IL-10, were detected in serum at 24 h after CI-RP (Figure 3). These results imply that interventions should be performed within 24 h following a stroke, before the levels of inflammatory cytokines increase.

**Figure 4:**
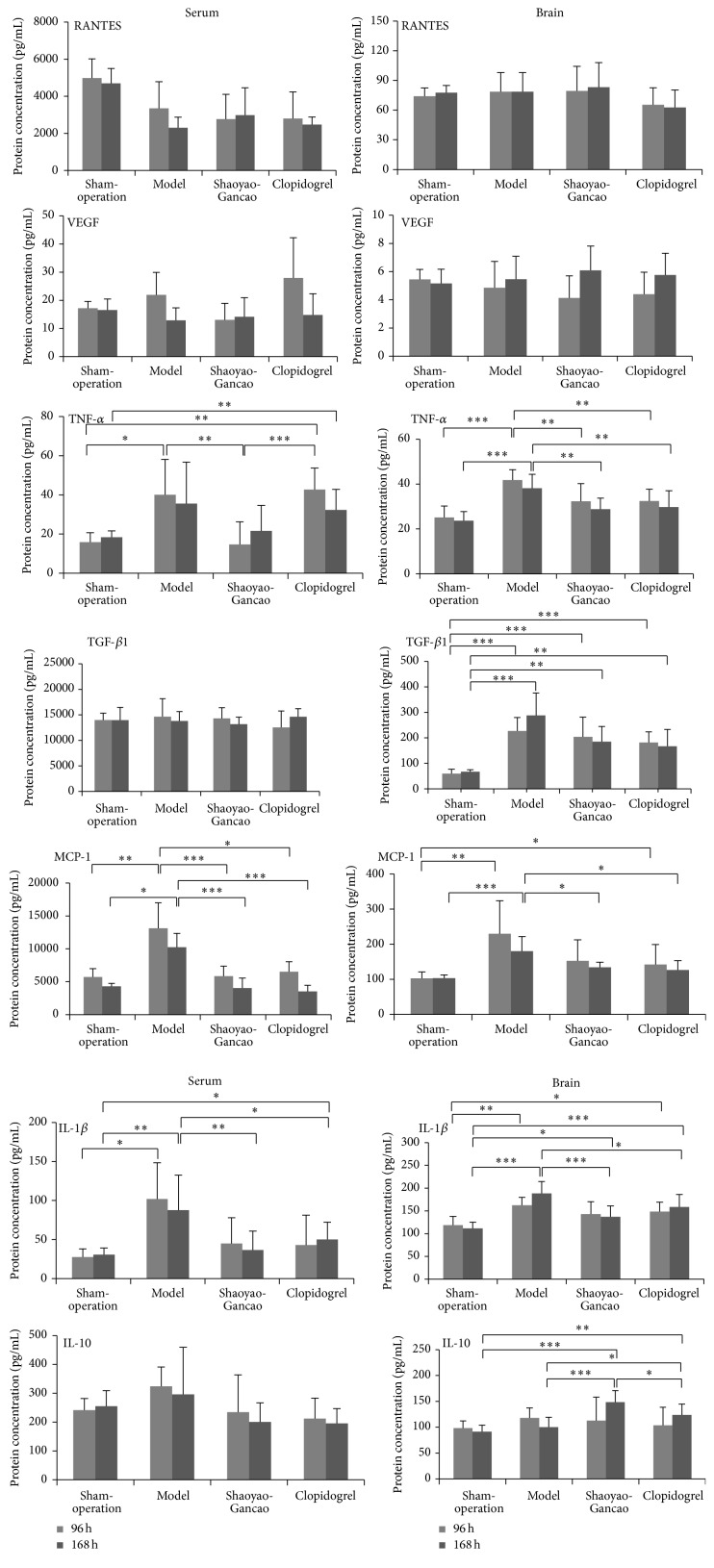
Changes in the levels of proinflammatory cytokines and angiogenic factors in the brain tissue and serum of rats at 96 and 168 h after CI-RP in the sham-surgery, model, SGD, and clopidogrel groups. ^*∗*^ *P* < 0.05; ^*∗∗*^ *P* < 0.01; ^*∗∗∗*^ *P* < 0.001.

Changes in the levels of inflammatory cytokine factors in brain tissue and serum were detected at 96 and 168 h after CI-RP in the sham-surgery, model, SGD, and clopidogrel groups (Figure 4). No significant effect of SGD or clopidogrel treatment on the level of RANTES was detected. The level of VEGF in brain tissue and serum at 96 h after CI-RP was not affected by treatment with SGD or clopidogrel.

However, the concentrations of TNF-*α* in serum and brain tissues from the affected sides were significantly increased (*P* < 0.05), but particularly in the SDG rats this increase was only visible in the brain to a smaller extent than in the model rats. These results suggest that SGD treatment significantly reduces the levels of TNF-*α* in the serum and brain following a stroke.

The level of TGF-*β*1 in the brain tissue from the affected side was increased after CI-RP (*P* < 0.001) but to a lesser extent in the SGD or clopidogrel treatment groups. The concentrations of MCP-1 in the serum and brain tissue of the model rats were increased after 96 h and 168 h after CI-IP, but these increases were significantly reduced by SGD or clopidogrel with a similar pattern of IL-1*β* serum but less profound in brain tissue concentrations.

The effects of SGD and clopidogrel on the level of IL-10 in the serum and brain tissue were also different. Treatment with SGD had no significant effect on the serum level of IL-10 but both SGD and clopidogrel significantly increased the level of IL-10 in brain tissue following CI-RP at 168 h after CI-RP (Figure 4).

### 3.2. Effect of SGD on Neurological Behavior in Rats after CI-RP

**Figure 5:**
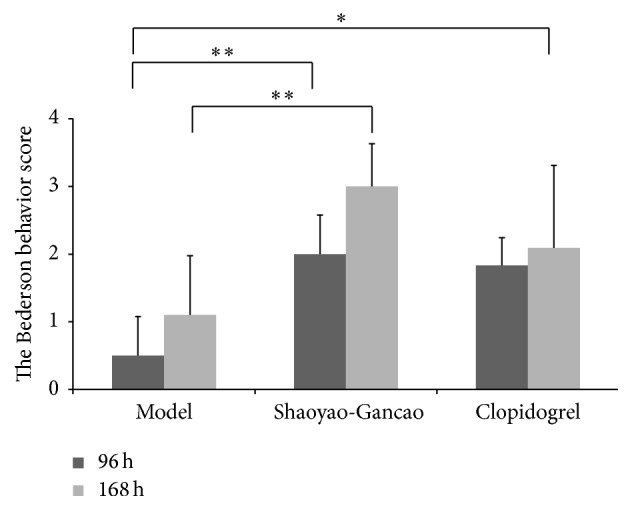
The Bederson scores for the model, SGD, and clopidogrel groups at 96 h and 168 h after CI-RP. ^*∗*^ *P* < 0.05; ^*∗∗*^ *P* < 0.01; ^*∗∗∗*^ *P* < 0.001.

The effects of SGD on the neurological behavior of rats after CI-RP were evaluated based on a modified Bederson scoring system. The Bederson scores for the SGD group at 96 and 168 h after CI-RP were higher (*P* < 0.01) than the score for the model group, whereas the Bederson score in the clopidogrel group was improved only at 96 h (*P* < 0.05) compared to the model rats. Though not statistically significant, the SGD group appeared to demonstrate better improvement in the Bederson score at 168 h than the clopidogrel group. These data indicated that SGD treatment was effective in improving behavioral dysfunction after CI-RP (Figure 5).

### 3.3. SGD Improved Neuronal Activity in Cortex and Peripheral Tissues after CI-RP

**Figure 6:**
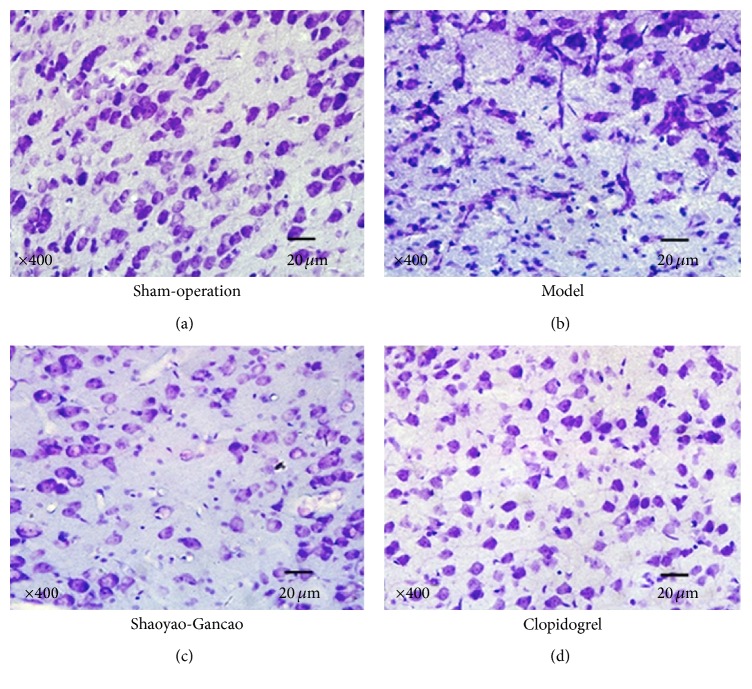
Nissl staining of brain tissue at 168 h after CI-RP shows both blue rounded Nissl bodies and cell nuclei in neurons.

After cresyl violet staining, blue round Nissl bodies and cell nuclei were identified in the neurons of the sham-surgery group. At 24 h after CI-RP, the outline of neurons was indistinct and the number of healthy neurons was reduced. Neurons were relatively smaller and nuclear pyknosis was clearly visible against the darkly stained cytoplasm. At 96 h after CI-RP, fewer healthy neurons were observed in all 3 groups, but nuclear pyknosis and darkly stained neurons were more abundant in the CI-RP model group. In the SGD group, a relatively large number of healthy neurons and nuclear pyknosis were observed, whereas healthy neurons, shrunken, and distorted neurons and dense darkly stained neurons were observed in the clopidogrel group (data not shown). At 168 h after CI-RP, fewer healthy neurons were observed in the model group, which mainly exhibited microglial cells and distorted condensed darkly stained cells, but, in the SGD and clopidogrel groups, a small proportion of healthy neurons and distorted cells displaying partial nuclear pyknosis were clearly visible (Figure 6).

**Figure 7:**
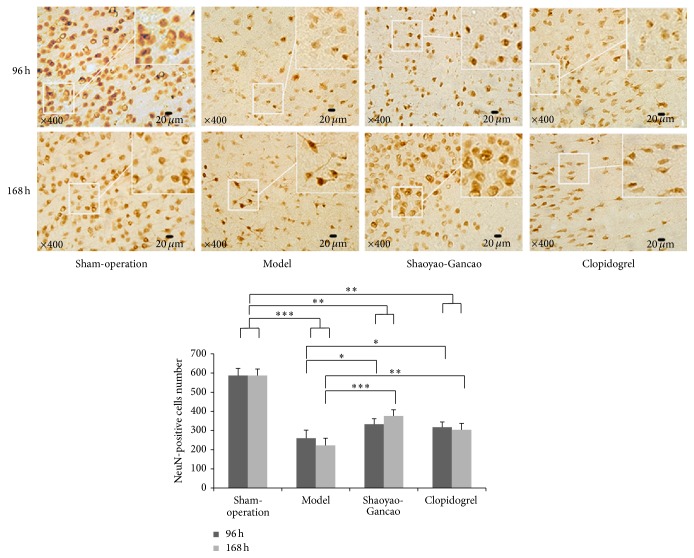
NeuN-positive cells in the infarcted cerebral cortex and peripheral tissues at the indicated time points after CI-RP. ^*∗*^ *P* < 0.05; ^*∗∗*^ *P* < 0.01; ^*∗∗∗*^ *P* < 0.001.

Immunohistochemical staining of NeuN, a protein specific to neurons, was used to identify neuronal death after CI-RP. In the sham-surgery group, many neurons with clear outlines, brown nuclei, clear structures, and protuberances were observed. At 96 h and 168 h after CI-RP, fewer neurons were observed in the model compared to the sham groups (*P* < 0.001), but at both time points the loss of neurons was significantly less in the SDG and clopidogrel groups (Figure 7).

**Figure 8:**
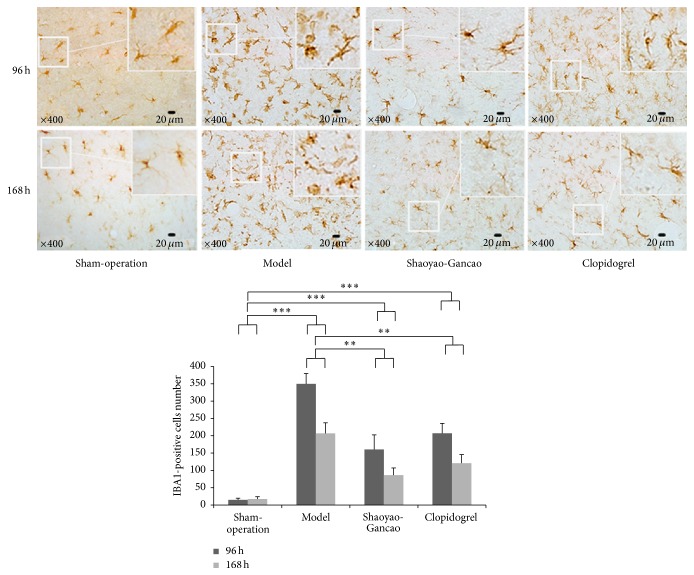
Microglial cell activation in the infarcted cerebral cortex and peripheral tissue at the indicated time points after CI-RP. ^*∗*^ *P* < 0.05; ^*∗∗*^ *P* < 0.01; ^*∗∗∗*^ *P* < 0.001.

Immunohistochemical staining of IBA1 was used to evaluate the activity of microglial cells in the infarcted cerebral cortex and peripheral tissues following CI-RP. In healthy cortical tissues, microglial cells were dark brown with small cell bodies and multiple slender branched protuberances. The activated microglial cells present in the infarcted and peripheral areas at 24 h after CI-RP had large cell bodies and less branches and exhibited darker staining. Of particular interest was the observation that some of the activated microglial cells were amoeboid. The number of amoeboid cells was increased at 96 h after CI-RP and gradually decreased thereafter until 168 h after CI-RP. The level of IBA1 expression was significantly lower in the SGD and clopidogrel groups at 96 h and 168 h after CI-RP than in the model group (*P* < 0.001), with the lowest level being observed in the SGD group after 168 h, but in all groups the number of IBA1-positive cells was significantly enhanced compared to the sham group (Figure 8).

**Figure 9:**
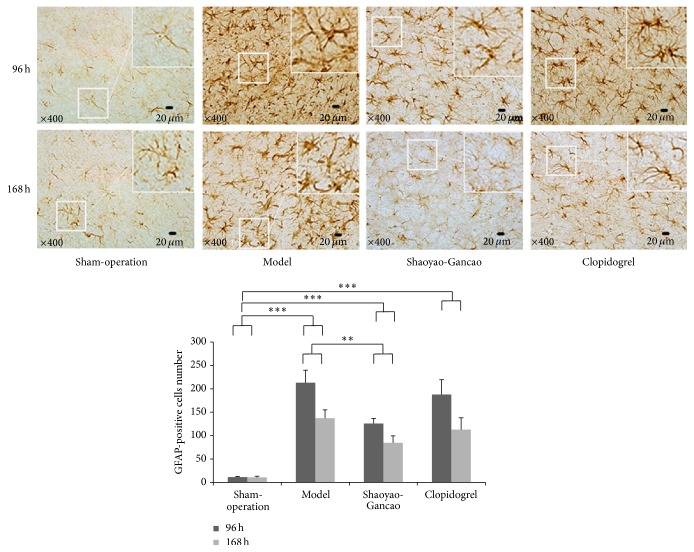
Characterization of astrocytes based on GFAP staining in the infarcted area of the cerebral cortex. ^*∗*^ *P* < 0.05; ^*∗∗*^ *P* < 0.01; ^*∗∗∗*^ *P* < 0.001.

We also quantified the number of astrocytes in the infarcted area of the cerebral cortex following CI-RP, based on immunohistochemical staining of GFAP, since astrocyte activation indicates brain trauma [26] (Figure 9). In healthy cortical tissue, astrocytes were sparse, had small cell bodies, and were only faintly stained. However, astrocytes in the infarcted area of the cerebral cortex were activated to a large degree, as indicated by dark brown GFAP staining, and had large cell bodies and thick protuberances. The numbers of activated astrocytes at 96 h and 168 h after CI-RP were significantly higher in the model compared to the sham group (*P* < 0.001).

The level of GFAP staining was also enhanced in the SDG and clopidogrel groups at 96 h and 168 h after CI-RP but in the SDG group to a significantly lower level (*P* < 0.01).

## 4. Discussion

The results of the present study showed that the expression levels of TNF-*α* and MCP-1 in serum and brain tissue at 24 h after CI-RP were significantly elevated compared with those in the sham-surgery control group. No significant changes in the levels of RANTES and VEGF were observed in brain tissue. However, in serum, the levels of RANTES and VEGF at 168 h after CI-RP were significantly different from those in the sham-surgery group. The expression levels of TGF-*β*1, IL-1*β*, and IL-10 in serum and brain tissues were also different (Figure 3). The changes in the expression patterns of proinflammatory cytokines in brain tissue and serum after CI-RP was dynamic, as differences in the expression of some of the cytokines and chemotactic factors in cerebral intrinsic cells were different after CI-RP [27, 28]. This is likely the result of different cells expressing the inflammatory cytokines and chemotactic factors in the brain after CI-RP. An adhesion factor for vascular endothelial cells, MCP-1, can induce circulating monocytes to invade brain tissue via the microvasculature resulting in high numbers of infiltrating neutrophils, monocytes, macrophages, and T cells [29, 30]. The serum level of MCP-1 at 24 h after CI-RP was 6 times higher than in the sham-surgery group and gradually declined over time whereas the level of MCP-1 in brain tissue increased over time.

The level of TGF-*β*1 in serum did not differ significantly from that of the control group at any time point after CI-RP (Figure 3). However, in brain tissue a significant positive linear correlation was observed at different time points after CI-RP, which was probably due to the production of TGF-*β*1 by neurons, astrocytes, microglial cells, and oligodendrocytes. A multifunctional cytokine, TGF-*β*1, is expressed at low levels in healthy brain tissue. Previous studies have shown that TGF-*β*1 can facilitate neuronal repair after cerebral ischemia injury [31]. The increased expression of TGF-*β*1 by astrocytes and microglial cells at 96 h and 168 h after CI-RP is consistent with the observed proliferation of astrocytes and microglial cells (Figures 8 and 9).

The expression patterns of IL-1*β* and TNF-*α* reflected the role of IL-1*β* in initiating the inflammatory response. Although TNF-*α* is an important inflammatory injury effector, high levels of TNF-*α* can also be cytotoxic. The most significant finding of our study was that the serum levels of IL-1*β*, MCP-1, and TNF-*α* had increased at 24 h after CI-RP. We also found that the dynamic changes in the levels of inflammatory cytokines in brain tissue following CI-RP were time-dependent. Compared with the findings of a previous study of the local inflammatory response after CI-RP [11], the increased serum levels of inflammatory cytokines originating from brain tissue provide a more comprehensive explanation of the role of the blood brain barrier in the inflammatory response following CI-RP.

In the present study, both SGD and clopidogrel inhibited the inflammatory response arising from cerebral infarct and our results demonstrated unequivocally that SGD was a more potent inhibitor of the expression of IL-1*β*, TNF-*α*, and MCP-1 than clopidogrel. The SGD- and clopidogrel-mediated reductions in TGF-*β*1 in brain tissue were likely associated with inhibition of the activation of microglial cells and astrocytes, which would also reduce the expression of TGF-*β*1. Following CI-RP, the inflammatory response was initiated in both brain tissue and serum, but the response in the serum primarily consisted of changes in the levels of proinflammatory cytokines, which is unlikely to cause substantial secretion of TGF-*β*1 by platelets [21].

Our results also showed that SGD and clopidogrel upregulated the expression of IL-10 in brain tissue, with the highest level of IL-10 observed in the SGD group. An important anti-inflammatory factor, IL-10, has been shown to produce neuroprotective effects [32]. Ooboshi et al. reported that the transfer of IL-10 to the lateral ventricle of rats after CI-RP reduced the size of the cerebral infarct and alleviated the inflammatory response [33]. In the present study, IL-10 expression in the brain was significantly upregulated at 168 h after CI-RP in rats treated with SGD or clopidogrel. Therefore, IL-10 likely suppresses the inflammatory response and promotes nerve regeneration. The serum level of IL-10 in the model group was highest at 24 h after CI-RP, whereas the level of IL-10 in the sham-surgery group dramatically declined after the 24 h time point. In contrast, treatment with SGD or clopidogrel had no effect on the serum level of IL-10. It is possible that SGD produces neuroprotective effects by affecting both proinflammatory and anti-inflammatory factors in brain tissue, which is consistent with the substantial improvements in the neurological behavior scores of rats in the SGD group at 96 h and 168 h after CI-RP.

We also examined the expression of RANTES and VEGF following CI-RP (Figures 3 and 4). The RANTES chemokine participates in a variety of biological actions including chemotaxis, the activation of lymphocytes, the regulation of cell growth and differentiation, and the formation of atherosclerotic plaques [34]. Previous studies of RANTES have primarily focused on its role in atherosclerosis, Alzheimer's disease, and AIDS, whereas there is a paucity of studies of its potential role in stroke. Our results have shown that the levels of RANTES in brain tissue did not change significantly after CI-RP and that the level of RANTES in the serum following CI-RP is relatively low. Overall, the SGD and clopidogrel interventions had no substantial effect on the levels of RANTES in brain tissue or serum, which suggests that it is not involved in the inflammatory response caused by CI-RP.

The expression of VEGF in macrophages, glial cells, and vascular endothelial cells has previously been shown to play a vital role in the response to focal cerebral ischemia and vascular proliferation in the cerebral ischemic penumbra [35]. In the model group, we found no change in VEGF levels after CI-RP and no VEGF enhancements were detected in the brain tissue of rats in the SGD and clopidogrel groups (Figure 4). However, serum levels of VEGF were significantly higher in the model group at 24 h after CI-RP, decreasing to normal levels at 168 h after CI-RP (Figure 3). Cerebral ischemia may trigger a stress reaction that causes the upregulation of VEGF expression in the serum, which will inhibit inflammation. The SGD-induced reduction in the levels of proinflammatory cytokines in brain tissue and serum after CI-RP demonstrates that SGD enhances the survival of cerebral cortical neurons. In the model group, we observed reductions in the number of neurons in the infarcted cerebral cortex and peripheral tissues at each time point following CI-RP and also that the rate of neuronal apoptosis increased over time. Both SGD and clopidogrel enhanced neuronal survival, but our results indicated that survival was improved by different mechanisms. Future studies are warranted to establish whether SGD and clopidogrel exhibit synergistic effects on infarcted cortical neurons when used in combination and to investigate whether different drugs can be used after CI-RP.

In conclusion, SGD effectively inhibited the activation of microglial cells and astrocytes after CI-RP and reduced the expression of IL-1*β*, TNF-*α*, and MCP-1 in brain tissue and serum, while upregulating the expression of IL-10. In rats treated with SGD, post-CI-RP neurological deficits were ameliorated and the survival of infarcted cortical neurons increased. Therefore, SGD may represent an effective treatment to reduce cerebral injury after stroke.
